# Physical Properties, Chemical Analysis, and Evaluation of Antimicrobial Response of New Polylactide/Alginate/Copper Composite Materials

**DOI:** 10.3390/md18120660

**Published:** 2020-12-21

**Authors:** Marcin H. Kudzin, Maciej Boguń, Zdzisława Mrozińska, Anna Kaczmarek

**Affiliations:** Lukasiewicz Research Network-Textile Research Institute, Brzezinska 5/15, 92-103 Lodz, Poland; mbogun@iw.lodz.pl (M.B.); zmrozinska@iw.lodz.pl (Z.M.); akaczmarek@iw.lodz.pl (A.K.)

**Keywords:** alginic acid, poly(lactide), copper, polymers, nonwoven fabric, melt-blown, composite, biodegradable, antibacterial activity

## Abstract

In recent years, due to an expansion of antibiotic-resistant microorganisms, there has been growing interest in biodegradable and antibacterial polymers that can be used in selected biomedical applications. The present work describes the synthesis of antimicrobial polylactide-copper alginate (PLA–ALG–Cu^2+^) composite fibers and their characterization. The composites were prepared by immersing PLA fibers in aqueous solution of sodium alginate, followed by ionic cross-linking of alginate chains within the polylactide fibers with Cu(II) ions to yield PLA–ALG–Cu^2+^ composite fibers. The composites, so prepared, were characterized by scanning electron microscopy (SEM), UV/VIS transmittance and attenuated total reflection Fourier-transform infrared spectroscopy ATR-FTIR, and by determination of their specific surface area (SSA), total/average pore volumes (through application of the 5-point Brunauer–Emmett–Teller method (BET)), and ability to block UV radiation (determination of the ultraviolet protection factor (UPF) of samples). The composites were also subjected to in vitro antimicrobial activity evaluation tests against colonies of Gram-negative (*E. coli*) and Gram-positive (*S. aureus*) bacteria and antifungal susceptibility tests against *Aspergillus niger* and *Chaetomium globosum* fungal mold species. All the results obtained in this work showed that the obtained composites were promising materials to be used as an antimicrobial wound dressing.

## 1. Introduction

Poly(lactic acid) (PLA) presents a polyester-type polymer [1,2], which due to its physicochemical properties and technical parameters [3,4], biodegradability and bioavailability [3,5,6,7,8,9,10,11] is dedicated to biomedical applications [12,13,14,15,16,17,18,19,20] These pro-medical attributes of PLA can be utilized in the form of its antibacterial composites, prepared on the PLA surface-modified matrix with antimicrobial additivities [3,8]. Copper and its salts play a special role in inorganic antimicrobials; there were nearly 5300 documents on antibacterial copper abstracted by Scopus [21]; it is vitally essential for many biological processes [22,23] and is a cheap and antibacterially-efficient inorganic [23].

Since the effective antibacterial composite should exhibit prolonged antibacterial activity, stable surface deposition/attachment of copper to polymer presents the major problem. Due to low affinity of metallic cations to carboxylic ester bonds [23], PLA weakly binds copper ions, and its antibacterial PLA-Cu composites require an interface covering layer with high affinity to copper.

Such requirements are fulfilled by alginates, marine-originated [24], biodegradable [25,26,27,28,29] biopolymers applied as a basis for drug delivery, wound dressings, and tissue engineering [30,31,32,33,34]. The strong affinity of alginates to metal cations allows for their application as antibacterial hybrids (e.g., [35,36,37,38,39,40,41,42,43,44]), and also for the removal of heavy metal salts, e.g., copper, lead, and mercury (e.g., [45,46,47,48,49,50]). The role of alginate in antibacterial finishing of textiles has been reviewed recently by Li et al. [51].

**Figure 1:**
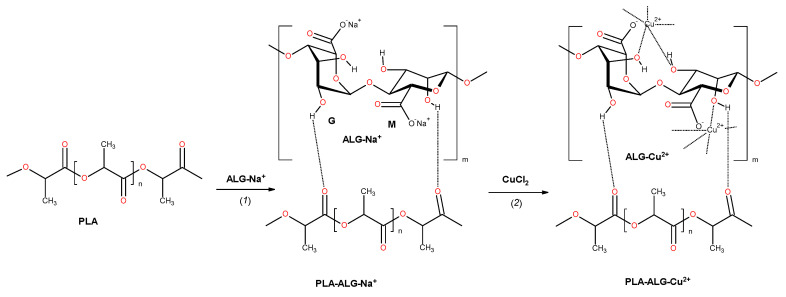
The reactions involved in the preparation of fibrous composite: PLA→PLA–ALG–Na^+^→PLA–ALG–Cu^2+^. The structure of alginate is presented as a linear copolymer –[GM]*_n_*– with homopolymeric blocks of (1–4)-linked β-d-mannuronate (M) and its C-5 epimer α-l-guluronate (G) residues.

We propose an application of alginate film covering the PLA matrix (PLA–ALG), which after addition of copper salts underwent cross-linking with formation of an outer-space coating, with strongly-fixed copper ions (Figure 1). Such PLA–ALG–Cu^2+^ nonwoven composites slowly release of copper ions providing long-termed antibacterial activity.

Is worth noting that cellulose-based fibers [52,53,54] and also wool fabric functionalized with copper alginate were reported to exhibit antibacterial properties [55]. However, in spite of the huge number reports on alginate abstracted by Scopus (12,643 reports) [56], or those on alginate composites (3450 reports) [57] or alginate hybrids (1237 reports) [58], the application of PLA–ALG hybrids has been reported in only a few papers, e.g., as a tissue engineering material [59], as composite microcapsules for a single-shot vaccine [60], and as bio-polymer carriers which can be implanted in subcutaneous tissue for continuous monitoring of glucose [61].

As part of our investigations focused on biologically-active phosphonates [62,63,64] and fibrous materials functionalization [65,66,67,68,69,70] we present the preparation and biological and physico-chemical properties of polylactide/alginate/copper composite materials.

## 2. Results and Discussion

### 2.1. Preparation of PLA–ALG–Cu^2+^ Composites

Sodium alginate with abundant carboxylate and hydroxyl groups [71], reacts with divalent cations such as Cu(II) [72], and others [73,74,75], to form cross-linked hydrogels, existing in reticular structures called “egg box” structures [76,77]. In transition metal-alginate systems, the sol-gel transition is characterized by a complex formation in which only the carboxyl groups in both M and G residues are coordinated to the metal ions [78]. In these structures, ALG-metal metal ions are bonded so strongly that alginates are used as metal sorbents for removal from environmental wastes [77,79,80,81,82]. Therefore, ALG–Cu^2+^ complexes, and subsequently PLA–ALG–Cu^2+^ composites are stable with a prolonged application period.

The preparation of PLA–ALG–Cu^2+^ composites wase performed by treating of PLA nonwovens with aqueous solutions of sodium alginate, during which alginate underwent adhesion to the PLA surface. In the second stage of the procedure, solution of copper chloride was added and consequently copper ions substituted sodium ions, and in turn initiated a cross-linking reaction with subsequent sol transition. The reactions involved in the fibrous composite preparation are depicted schematically in Figure 1.

### 2.2. Scanning Electron Microscopy

**Figure 2:**
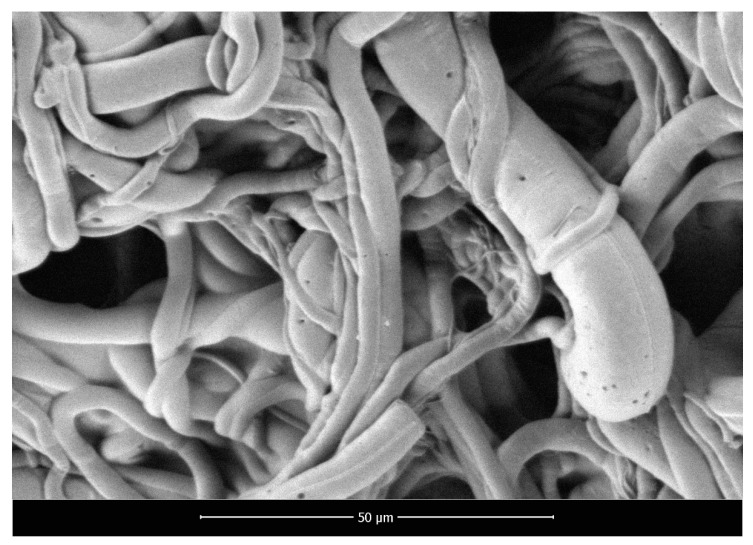
Scanning electron microscopy images of PLA nonwoven, magnification: 2000×.

**Figure 3:**
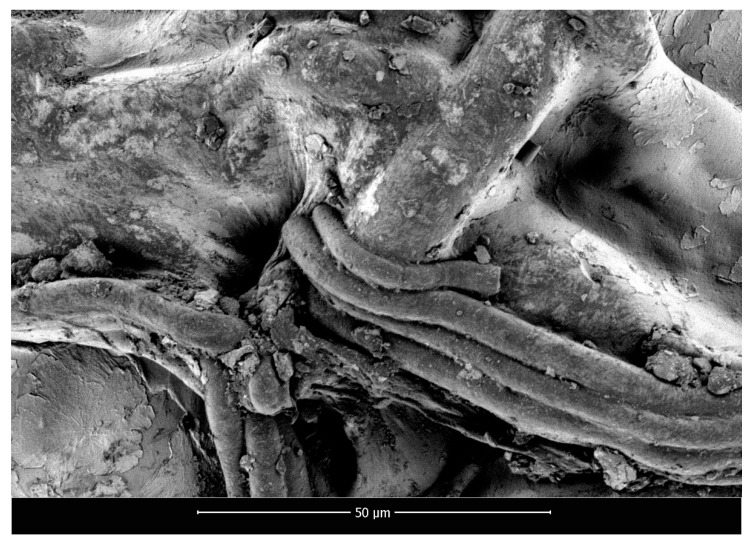
Scanning electron microscopy images of PLA–ALG–Na^+^, magnification: 2000×.

**Figure 4:**
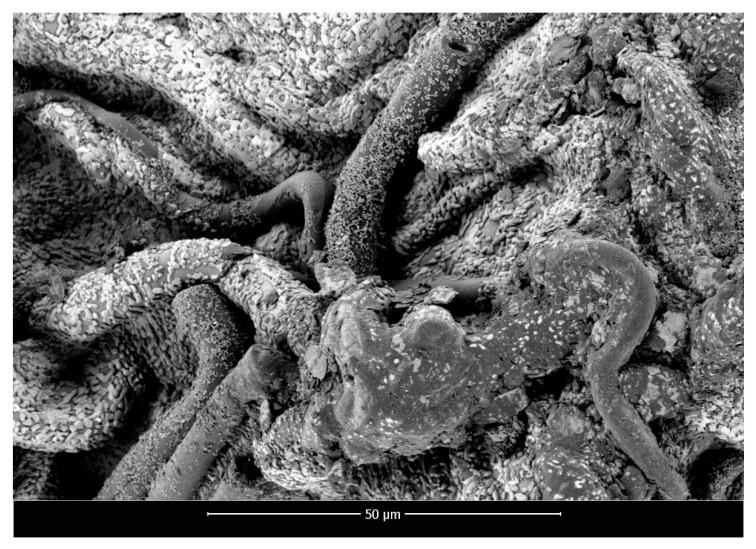
Scanning electron microscopy images of PLA–ALG–Cu^2+^-2, magnification: 2000×.

SEM micrographs of polylactide nonwoven (PLA), polylactide/sodium alginate (PLA–ALG–Na^+^), and PLA–ALG–Cu^2+^-2 composites are presented in Figure 2, Figure 3 and Figure 4, respectively.

The presented SEM images illustrate the changes in the surface morphology of the investigated samples occurring due to the modification of PLA nonwovens with the solution of alginic acid sodium salt and CuCl_2_. The SEM image of unmodified PLA nonwoven presents a mesh of randomly-oriented fibers, with interconnected pores and relatively-smooth surface (Figure 2). The diameters of the fibers vary significantly from approximately 0.7 µm up to 14.0 µm. Surface modification of PLA nonwovens with the alginate resulted in the occurrence of the coating on the surface of the randomly-oriented fibers (Figure 3). As a result, fewer pores are visible. In addition, the coating covers thinner fibers and only fibers with a diameter greater than ~3.5 µm can be distinguished. Furthermore, the agglomerates of alginate may be noticed on the surface of the fibrous composite. Due to that, the surface of PLA–ALG–Na^+^ fibers is more coarse in comparison to unmodified PLA fibers.

Figure 4 illustrates the surface of the PLA–ALG–Cu^2+^-2 composite (charged with ~7.6% of Cu^2+^), with the agglomerates, presumably composed of ALG–Cu^2+^, visible over the entire surface of the sample. Similarly as in the case of the PLA–ALG–Na^+^ sample, only the fibers with the diameter higher than ~4. µm are distinguishable and the majority of pores are covered by the coating. Moreover, the surface of the PLA–ALG–Cu^2+^ composite is less uniform and rougher due to the presence of numerous agglomerates.

**Figure 5:**
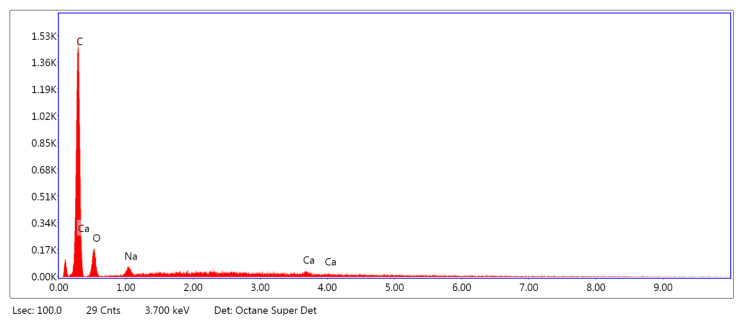
Example of the energy-dispersive X-ray Spectroscopy (EDS) spectrum of PLA–ALG–Na^+^.

**Figure 6:**
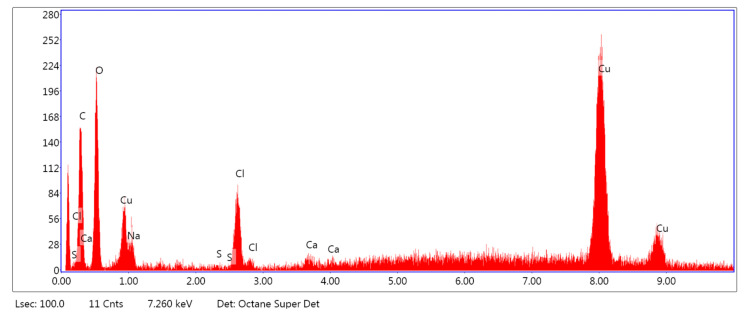
Example of the EDS spectrum of the PLA–ALG–Cu^2+^-1.

**Figure 7:**
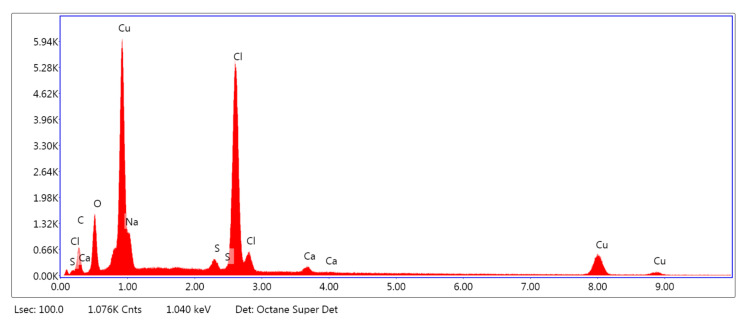
Example of the EDS spectrum of PLA–ALG–Cu^2+^-2.

Figure 5, Figure 6 and Figure 7 show the exemplary EDS spectra of PLA–ALG–Na^+^ (Figure 5), PLA–ALG–Cu^2+^-1 (Cu: 1.4%) (Figure 6) and PLA–ALG–Cu^2+^-2 (Cu: 7.6%) (Figure 7) composites (the EDS data are presented as a graph with energy (keV) on the *x*–axis and peak intensity on the *y*–axis). Table 1 presents the chemical composition of the investigated samples obtained from the quantitative analysis of EDS results. The presented values of mean concentration of each element are calculated from six to eight spot measurements, on two different samples.

As can be observed from Figure 5, the PLA–ALG–Na^+^ sample was composed primarily from carbon, oxygen, and sodium. This is consistent with the chemical composition of PLA, which is built of carbon, oxygen, and hydrogen. The occurrence of a sodium peak may be attributed to the presence of alginate, which apart from carbon, oxygen, and hydrogen also contains sodium. In the case of the PLA–ALG–Cu^2+^-1 and PLA–ALG–Cu^2+^-2 composites, additional peaks associated with copper and chloride were detected. This confirms the presence of NaCl (ALG–Na^+^ + Cu^2+^ + 2Cl^−^ → ALG–Cu^2+^ + 2Na^+^ + 2Cl^−^) in those samples. Apart from that, the calcium peak was observed for all the samples. Moreover, in the case of fibrous composites containing NaCl a peak related to sulfur was also noticed. The relative intensity of the sulfur peak increased with the NaCl concentration in the surface modifier.

Quantitative EDS analysis (Table 1) exhibited that the modification of PLA with alginate led to an increase in carbon concentration (from 51.70 at. % to 71.41 at. %). Simultaneously, a decrease in the oxygen content (from 48.33 at. % to 28.42 at. %) was observed. Additionally, for the PLA–ALG–Na^+^ composite a small concentration of sodium was detected (0.10 at. %). Furthermore, the obtained results indicate that the content of Cu and Cl significantly increased when the concentration of CuCl_2_ in the surface modifier changed from 5% to 10%. In the case of the PLA–ALG–Cu^2+^-1 sample, the content of copper was 0.92 at. %, while the chloride concentration was 1.93 at. % (Figure 5). For the PLA–ALG–Cu^2+^-2 composite the concentrations of copper and chloride rose to 15.06 at. % and 21.44 at. %, respectively (Figure 6). At the same time, with the increase of CuCl_2_ concentration from 5% to 10%, the carbon content dropped to 55.25 at. % and 35.16 at. %, while sodium content increased to 0.78 at. % and 5.87 at. %, accordingly. In the case of oxygen concentration, at first an increase of up to 40.95 at. % was observed for the PLA–ALG–Cu^2+^-1 sample, followed by a decrease to 19.96 at. % for the PLA–ALG–Cu^2+^-2 composite. As far as calcium and sulfur contents were concerned, both rose with the increase in the CuCl_2_ concentration, however, the observed values did not exceed 1.5 at. % and thus, may be regarded as a contamination. The observed high standard deviation values indicates that the distribution of chemical components was not uniform and homogeneous.

### 2.3. FAAS

**Figure 8:**
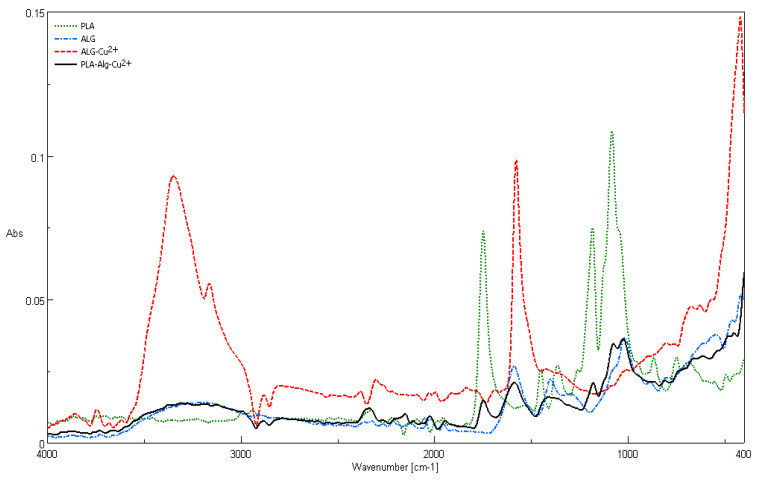
Comparison of attenuated total reflection Fourier–Transform infrared spectroscopy ATR–FTIR spectra of PLA nonwovens, alginic acid sodium salt (ALG–Na^+^), ALG–Cu^2+^ complex, and PLA–ALG–Cu^2+^-2 composite.

The copper content in the PLA–ALG–Cu^2+^ composites was assessed by the flame atomic absorption spectrometry (FAAS) method, after prior composite degradation, as shown in Figure 8. The results are given in Table 2 [83].

The results of FAAS analysis showed that copper content in the fibrous composites samples depends on the concentation of water solution of copper(II) chloride in the second step of a dip-coating modification. The higher coating concentration of the copper(II) chloride modifier (10%) gave the higher content of the Cu^2+^ on PLA sample (73.91 g/kg).

### 2.4. ATR–FTIR Spectra

The recorded ATR–FTIR spectra for polylactide nonwoven, alginic acid sodium salt (ALG–Na^+^), ALG–Cu^2+^ complex, and PLA–ALG–Cu^2+^-2 composite are presented in Figure 8. Characteristic FTIR signals of the composite and its components (PLA, ALG–Na^+^, and ALG–Cu^2^) are summarized in Table 3 [84,85,86,87,88,89,90,91,92].

The infrared vibrations spectrum of PLA–ALG–Cu^2+^ composite contains the major bands derived from the composite components, namely PLA (1760 cm^−1^ (ν C=O), 1073 cm^−1^ (ν_s_ C–O–C), and 866 cm^−1^ (ν C–CO_2_^−^)), ALG–Na^+^ /ALG–Cu2+ (3200 cm^−1^ (ν_s_ O–H), 2890 cm^−1^ (ν_s_ C–H), 2337 cm^−1^, 2152 cm^−1^, 1158 cm^−1^, 1417 cm^−1^ (δ C–H, ν C–CO_2_^−^), 1073 cm^−1^, 1020 cm^−1^, 801 cm^−1^, and 555 cm^−1^ (δ_ω_ O–H)).

Some bands characteristic for PLA or ALG–Na^+^ disappeared, due to an overlapping effect or a band broadening caused by influence of cupric ion, for instance in PLA spectrum (2997 cm^−1^ (ν_as_ CH_3_), 2947 cm^−1^ (ν_s_ CH_3_), 1452 cm^−1^ (δ_as_ CH_3_), 1388–1368 cm^−1^ (δ CH^+^, δ_s_ CH_3_), 1270 cm^−1^ (δ CH^+^, ν C–O–C), 1045 cm^−1^ (ν C–CH_3_), 875–860 cm^−1^ (ν C–CO_2_^–^), 760–740 cm^−1^ (δ C=O), and 715–695 cm^−1^ (γ C=O)).

### 2.5. Specific Surface Area, Total Pore Volume, and Average Pore Diameter Measurement

The specific surface area, total pore volume, and average pore diameter of the PLA–ALG–Cu^2+^ composites samples are presented in Table 4.

The specific surface area of the unmodified PLA nonwoven was equal to 0.2405 m^2^/g. The modification of the PLA nonwoven with the solution of alginic acid sodium salt resulted in ~130% increase in the value of specific surface area (up to 0.5548 m^2^/g). At the same time, the addition of 5% and 10% of CuCl_2_ caused even further growth of the specific surface area up to 0.8429 and 1.4280 m^2^/g, respectively. When compared to the PLA–ALG–Na^+^ sample, the specific surface area of PLA–ALG–Cu^2+^-1 sample rose by ~150%, while in the case of the PLA–ALG–Cu^2+^-2 sample an ~260% increase in the specific surface area value was observed. Thus, it can be concluded that the modification of PLA nonwovens with alginate and CuCl_2_ leads to a significant growth of the specific surface area. The higher CuCl_2_ content, the greater specific surface area.

The increase in the specific surface area observed for the modified nonwovens may be associated with the higher mesoporosity as indicated by the larger hysteresis loops for the adsorption–desorption isotherms of modified samples. This is confirmed by the total pore volume, which significantly rose from 9.084⋅10^−4^ cm^3^/g for the PLA nonwoven up to 1.581⋅10^−3^ cm^3^/g (170%), 3.450⋅10^−3^ cm^3^/g (380%) and 4.661⋅10^−3^ cm^3^/g (510%) for the PLA–ALG–Na^+^, PLA–ALG–Cu^2+^-1, and PLA–ALG–Cu^2+^-2 samples, respectively. It is worth highlighting, that despite the observed covering of pores visible in the SEM images of the modified samples, the mesoporosity of those samples was greater.

At the same time, there was no correlation between the specific surface area or total pore volume and the average pore diameter. The estimated average pore diameter of the investigated samples varied in the range of 10.54–16.59 nm and did not explicitly depend on the modification and CuCl_2_ concentration.

### 2.6. UV-VIS Analysis and Determination of the Protective Properties against UV Radiation

**Figure 9:**
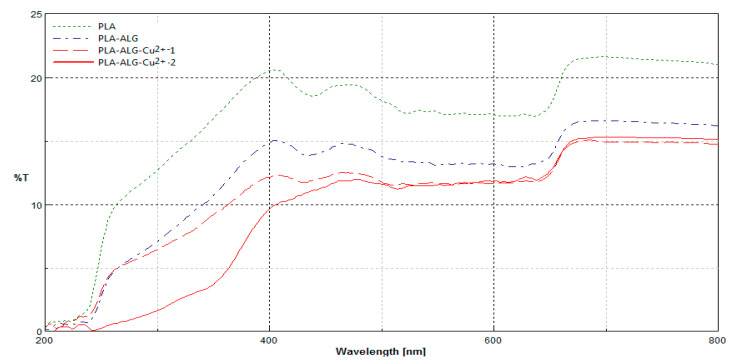
Comparison of transmittance spectra (%T) of PLA nonwoven and PLA–ALG–Na^+^ and PLA–ALG–Cu^2+^ (PLA–ALG–Cu^2+^-1 and PLA–ALG–Cu^2+^-2) composites, recorded in the range: λ = 200–800 nm.

Figure 9 presents spectrophotometric transmittance spectrum in the wavelength λ = 200–800 nm of PLA nonwoven and PLA–ALG–Na^+^ and PLA–ALG–Cu^2+^ (PLA–ALG–Cu^2+^-1 and PLA–ALG–Cu^2+^-2) composites.

The transmittance (%T) spectra in the range λ = 200–800 nm of the PLA and its composites (PLA–ALG–Na^+^ and PLA–ALG–Cu^2+^) revealed changes in their macrostructures in comparison to PLA nonwoven, expressed by a decrease in transmittance in all ranges of measurement. The unmodified (PLA) and modified samples (PLA–ALG–Na^+^, PLA–ALG–Cu^2+^ (PLA–ALG–Cu^2+^-1 and PLA–ALG–Cu^2+^-2) had similar spectral characteristics across the entire spectral range. The reduction in spectral transmission (PLA–ALG–Na^+^, PLA–ALG–Cu^2+^ (PLA–ALG–Cu^2+^-1 and PLA–ALG–Cu^2+^-2) was caused by an additional layer of alginate coating on the surface of the samples. There was a noticeable influence of the Cu content in a modified sample (PLA–ALG–Cu^2+^ (PLA–ALG–Cu^2+^-1 and PLA–ALG–Cu^2+^-2)) on a transmission level, especially in the range of a 250–500 nm–decrease of transmission level in the sample PLA–ALG–Cu^2+^-2 vs. PLA–ALG–Cu^2+^-1.

Table 5 compare average transmittance (T%) and calculated UPF values of modified samples PLA–ALG, PLA–ALG–Cu^2+^ (PLA–ALG–Cu^2+^-1 and PLA–ALG–Cu^2+^-2) with those of non–modified samples (PLA and PLA–ALG–Na^+^).

Very good barrier properties against UV radiation were obtained for PLA–ALG–Cu^2+^-2 (UPF = 43.28). This result indicates that the modification performed imparts proper barrier properties against UV radiation according to EN 13758-1:2002 [93].

## 3. Antimicrobial Properties

### 3.1. Antibacterial Activity

**Figure 10:**
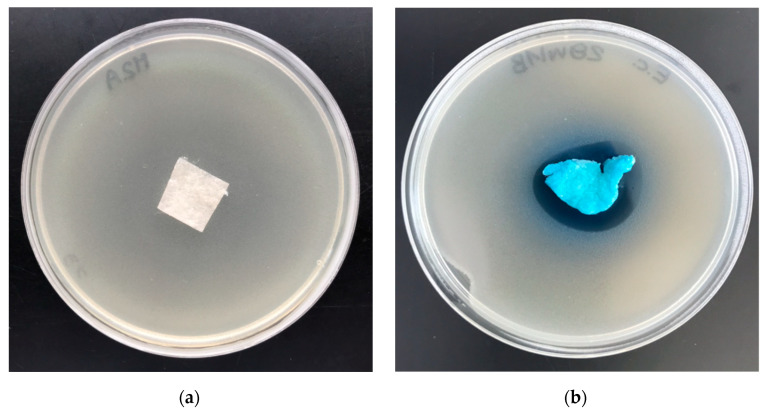
The polylactide/alginate/copper (PLA–ALG–Cu^2+^) composites’ antimicrobial activity tests. Inhibition zones of *Escherichia Coli* bacterial growth on Petri dishes, sample: (**a**) PLA and (**b**) PLA–ALGCu^2+^−2.

**Figure 11:**
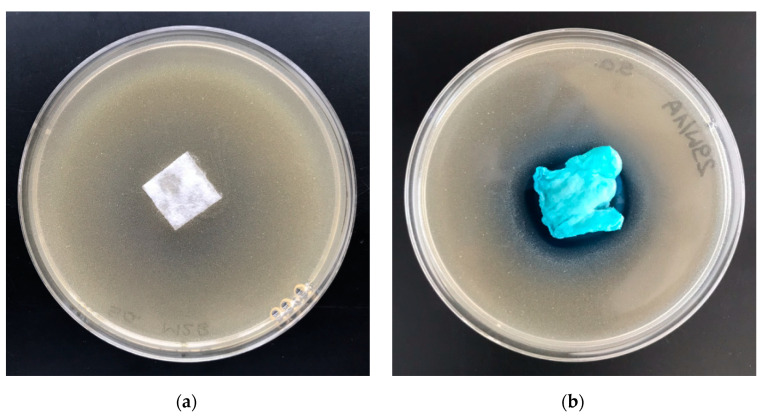
The polylactide/alginate/copper (PLA–ALG–Cu^2+^) composites’ antimicrobial activity tests. Inhibition zones of *Staphylococcus aureus* bacterial growth on Petri dishes, sample: (**a**) PLA and (**b**) PLA–ALG–Cu^2+^−2.

The polylactide/alginate/copper (PLA–ALG–Cu^2+^) composites were subjected to antimicrobial activity tests against Gram-negative *Escherichia coli* (ATCC11229) and Gram-positive *Staphylococcus aureus* (ATCC 6538) bacteria. Results of biological activity tests are listed in Table 6 and illustrated in Figure 10 and Figure 11.

Results of these studies prove antimicrobial protection against different bacterial microorganisms of functionalized materials (PLA–ALG–Cu^2+^) for *Escherichia* *coli* and *Staphylococcus* *aureus* (Table 6), expressed by strong, visible inhibition zones of bacterial growth on Petri dishes (Figure 10b and Figure 11b). A higher ability to inhibit the bacterial growth of *Staphylococcus aureus* was exhibited by the PLA–ALG–Cu^2+^-2 samples. Inhibition zones of bacterial growth of 3–4 mm and no bacterial growth in the samples tested confirmed this observation.

### 3.2. Antifungal Activity

**Figure 12:**
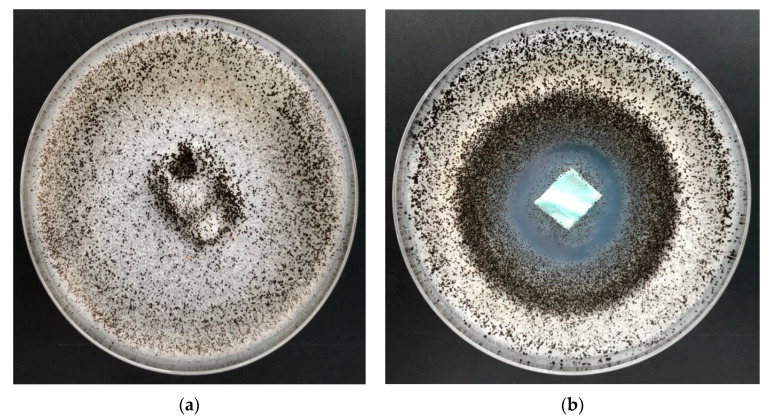
The PLA–ALG–Cu^2+^ composites antimicrobial activity tests against *Aspergillus niger* (ATCC 6275). Inhibition properties of fungal growth on Petri dishes: (**a**) PLA and (**b**) PLA–ALG–Cu^2+^−2.

**Figure 13:**
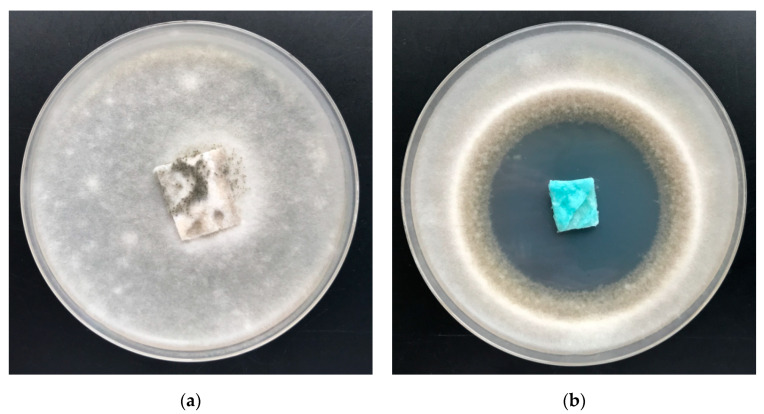
The PLA–ALG–Cu^2+^ composites’ antimicrobial activity tests against *Chaetomium globosum.* Inhibition properties of fungal growth on Petri dishes: (**a**) PLA and (**b**) PLA–ALG–Cu^2+^−2.

Results of antifungal activity tests in accordance with the PN EN 14119: 2005 point 10.5 (B2) against a colony of *Aspergillus niger* and *Chaetomium globosum* (ATCC 6205) of PLA fabrics and PLA–ALG–Cu^2+^ composites are illustrated in Figure 12 and Figure 13 and listed in Table 7 [95].

Non–modified samples of polylactide nonwoven (PLA) as well as alginate–modified polylactide nonwoven (PLA–ALG–Na^+^) did not inhibit the growth of *Aspergillus niger* or of *Chaetomium globosum* fungus, either around the samples or in the contact zone with the culture media. In addition, strong fungal growth covering of the entire surface of the samples was visible (Figure 12a and Figure 13a).

Antifungal activity was demonstrated by the PLA–ALG–Cu^2+^ composites samples and the results revealed strong visible inhibition zones of fungal growth on Petri dishes (Figure 12b and Figure 13b), and therefore proved antifungal protection against *Aspergillus niger* and *Chaetomium globosum*.

## 4. Conclusions

Medicine, especially at present, is focused on the search for new and more effective methods of combating microbials, especially multi-drug-resistant pathogens (viruses, bacteria, and fungi). In recent years there has been growing interest in biodegradable, “eco–friendly” and multifunctional polymers that can be used in selected biomedical applications. This paper presents a method for charging of poly(lactide) nonwoven fabrics with copper salt, and in this way making equipment with antibacterial activity. This was achieved by a two–step procedure of coating of PLA fibers with, prepared in situ, copper alginate (PLA→ PLA–ALG–Cu^2+^).

The structural properties of these new products were characterized by scanning electron microscopy (SEM), energy-dispersive spectroscopy (EDS), attenuated total reflectance Fourier-transform infrared (ATFR-IR) and ultraviolet-visible spectrophotometry (UV-Vis), and specific surface area, total pore volume and average pore diameter measurement. Copper content in the composites was determined by FAAS.

The results revealed that PLA–ALG–Cu^2+-^2 with ca. 7.4% copper in the composite, presents less uniform and rougher nonwoven in comparison with PLA, with diameter range of 4 to 6 µm, and a majority of pores covered by the coating (PLA–ALG–Cu^2+-^2 vs. PLA), and spotted with the agglomerates, presumably also ALG–Cu^2+^.

Specific surface area, total pore volume, and average pore diameter of the examined samples presented ~6-fold increase, ~2-fold decrease and similar average pore diameters for PLA–ALG–Cu^2+^-2 vs. PLA, respectively.

The transmittance (%T) spectra in the range λ = 200–800 nm of the PLA nonwoven, PLA–ALG–Na^+^ and PLA–ALG–Cu^2+^ composites revealed a decrease in transmittance in all range of measurement and very good barrier properties against UV radiation were obtained for PLA–ALG–Cu^2+^-2 (UPF = 43.28). 

The ATR–FTIR spectra of PLA–ALG–Cu^2+^ composites contained the major bands derived from the composite components, namely PLA (1760 cm^−1^ (ν C=O), 1073 cm^−1^ (νs C–O–C) and 866 cm^−1^ (ν C–CO_2_^–^)), ALG–Na^+^/ALG–Cu^2+^ (3200 cm^−1^ (νs O–H), 2890 cm-1 (νs C–H), 2337cm^−1^, 2152cm^−1^, 1158cm^−1^, 1417cm^−1^ (δ C–H, ν C–CO_2_^–^), 1073 cm^−1^, 1020 cm^−1^, 801 cm^−1^, and 555 cm^−1^ (δω O–H)).

The application benefits of the polylactide/alginate/copper materials were confirmed by antimicrobial tests against *Chaetomium globosum, Aspergillus niger* fungus species, and representative Gram–negative (*E. coli)* and Gram–positive (*S. aureus)* bacteria. 

In summary, PLA–ALG–Cu^2+^ is composed of two easily–biodegradable composites (PLA and ALG) and the entire procedure fulfills the requirements of eco–friendly standards, due to the exhibited technical properties and significant antimicrobial action that can find an application in different fields of medical or healthcare industry (e.g., air filtration, bioadhesives, and tissue engineering). The performed study allows us to state that modification of poly(lactide) nonwoven fabric using alginic acid and copper (II) chloride offers the possibility of additional functions and the creation of new added-value materials. Further studies will focus on the determination of mechanical and cytotoxicity properties of PLA-alginate composites.

## 5. Materials and Methods

### 5.1. Materials

- Poly(lactic acid) (PLA) type Ingeo™ Biopolymer 3251D, in the form of granulates was purchased from NatureWorks LLC (Minnetonka, MN, USA), T_mp_ = 160–170 °C, MFR = 30–40 g/10min (190 °C/2.16 kg) and was used for the forming of samples of nonwoven fabrics;
- Alginic acid sodium salt (CAS Number 9005-38-3, the molecular weight: 120,000–190,000 g/moL, mannuronic acid to guluronic acid–M/G ratio: 1.56) from Millipore Sigma (St. Louis, MO, USA), was used for surface modification of polymer nonwovens;
- Copper(II) chloride, CuCl_2_, 97% (CAS Number: 7447-39-4) from Millipore Sigma (St. Louis, MO, USA) was used for surface modification of nonwoven composite;
- Bacterial strains: *Escherichia coli* (ATCC 25922) and *Staphylococcus aureus* (ATCC 6538) were purchased from Microbiologics (St. Cloud, MN, USA).
- Fungal strains: *Aspergillus niger* van Tieghem (ATCC 6275) and *Chaetomium globosum* (ATCC 6205) were purchased from Microbiologics (St. Cloud, MN, USA).

### 5.2. Methods

#### 5.2.1. Nonwoven Fabrics

Nonwovens were fabricated by the melt-blown technique using a one-screw laboratory extruder (Axon, Limmared, Sweden) with a head with 30 holes of 0.25 mm diameter each, compressed air heater and collecting drum. The melt-blown process conditions applied for preparation of PLA nonwovens are presented in Table 8.

#### 5.2.2. Dip–Coating Modification

Nonwoven samples (10 cm × 10 cm; 2.00 ± 0.05 g) were modified by the dip-coating, two-step method, impregnated in the solution of sodium alginate (step 1) and immersed in the solution of copper(II) chloride (step 2). An aqueous solution of sodium alginate (0.5%, 5 g/L) was homogeneously dispersed and had appropriate viscosity (about 50–70 dPas). An additional two different water solutions of copper(II) chloride was prepared—5 and 10%. The nonwoven fabric samples were impregnated in the polysaccharide solution for 1 min, then the samples were immediately transferred to one of the two variants of the aqueous copper(II) chloride solution (5 or 10%) and immersed in the solution for 1 min. Then the samples were squeezed and dried for 5 h at 50 °C (to a constant weight of 2.5 ± 0.05 g). After this modification, the formed nonwoven composites, further referred to as PLA–ALG–Cu^2+^-1 (5% CuCl_2_) or PLA–ALG–Cu^2+^-2 (10% CuCl_2_), respectively, or PLA–ALG–Na^+^ for a composite of PLA and coating paste without CuCl_2_, presented a visually–uniform, homogeneous structure. The components of the modifier used and the corresponding modifier abbreviations are given in Table 9.

#### 5.2.3. Scanning Electron Microscopy/Energy–Dispersive X–ray Spectroscopy (SEM/EDS)

The microscopic analysis of fibers was performed on a Tescan Vega 3 scanning electron microscope (Brno, Czech Republic). Magnification was from 20,000×. 

#### 5.2.4. Flame Atomic Absorption Spectrometry (FAAS)

**Figure 14:**
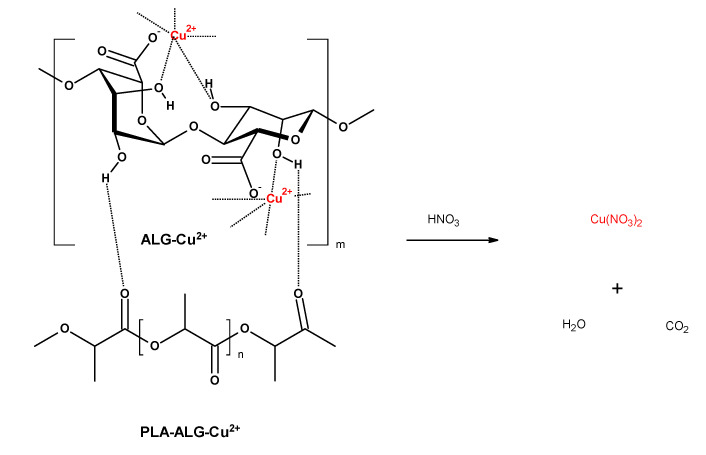
Mineralization of PLA–ALG–Cu^2+^.

Determination of copper content in PLA–ALG–Cu^2+^ composites was assessed by prior sample mineralization (Figure 14), using single-module Magnum II microwave mineralizer from Ertec (Wroclaw, Poland), as described earlier [70].

Determination of copper (II) ions was performed by atomic absorption spectrometry with flame excitation using Thermo Scientific Thermo Solar M6 (LabWrench, Midland, Canada) spectrometer equipped with a 100-mm titanium burner, coded lamps with a single-element hollow cathode, background correction: D2 deuterium lamp, as described earlier [65].

The total copper content *M* (mg/kg; ppm) in the PLA–ALG–Cu^2+^ composite sample was calculated according to the Equation (1) [83]:

(1) $$M=\frac{C\;\cdot \;V\;}{m}$$

where:

*C*—metal concentration in the mineralized PLA–ALG–Cu^2+^ sample solution (mg/L);

*m*—mass of the mineralized sample of PLA–ALG–Cu^2+^ composites (g);

*V*—volume of the sample solution (mL).

#### 5.2.5. Attenuated Total Reflection Fourier–Transform Infrared Spectroscopy (ATR–FTIR)

An analysis of the chemical surface structure of the of PLA–ALG–Cu^2+^ fibrous composite samples was performed by ATR–FTIR spectroscopy using a Jasco 4200 spectrometer (Tokyo, Japan) with an Pike Gladi ATR attachment (Cottonwood, AZ, USA) in the range of 400–4000 cm^−1^.

#### 5.2.6. Specific Surface Area, Total Pore Volume, and Average Pore Diameter Measurement

The specific surface area of the investigated samples was measured using the Autosorb-1 (Quantachrome Instruments, Boynton Beach, FL, USA) apparatus. The analysis was performed using the physisorption method with nitrogen used as a sorption agent. The measurements were carried out at 77 K. For each experiment, about 1–2 g of a given sample was weighed and used. Prior to the analysis, the samples were dried in 105 °C for 24 h and degassed overnight at room temperature. 

In order to determine the specific surface area, the 5-point Brunauer–Emmett–Teller (BET) method was applied. The specific surface area was calculated twice for each sample, using the 5-point adsorption isotherm (P/P_0_ in the range of 0.10–0.30) and the 39-point adsorption-desorption isotherm. The total pore volume and average pore diameter were determined from the 39-point adsorption-desorption isotherm (P/P_0_ in the range of 0.05–1.00). For that purpose, a single point at P/P_0_ ≈ 1.00 was analyzed.

#### 5.2.7. UV-VIS Analysis and Determination of the Protective Properties against UV Radiation

Changes of the physical properties as transmittance (%T) of samples occurring during modifications were assessed using double beam Jasco V-550 UV-VIS spectrophotometer (Tokyo, Japan) with integrating sphere attachment in the range of 200–800 nm, analogously as we described earlier [65]. The same apparatus was used to determine the ultraviolet protection factor (UPF) of samples.

The UPF value of the samples was determined, according to EN 13758-1:2002 standard [93], as the arithmetic mean of the UPF values (Equation (2)) for each of the samples (a confidence interval of 95%), analogously, as described earlier [67].

(2) $$UPF=\frac{\int_{290}^{400}E\left(\lambda \right)\varepsilon \left(\lambda \right)d\left(\lambda \right)}{\int_{290}^{400}E\left(\lambda \right)\varepsilon \left(\lambda \right)T\left(\lambda \right)d\left(\lambda \right)}$$

where:

*E*(*λ*)—the solar irradiance;

*ε*(*λ*)—the erythema action spectrum (measure of the harmfulness of UV radiation for human skin);

Δ*λ*—the wavelength interval of the measurements;

*T*(*λ*)—the spectral transmittance at wavelength *λ*.

#### 5.2.8. Antibacterial Activity

The PLA–ALG–Cu^2+^ fabrics’ anti-bacterial activity was tested according to standard: PN-EN ISO 20645:2006 [94], against a colony of Gram-positive bacteria, *Staphylococcus aureus* (ATCC 6538) and Gram-negative bacteria, *Escherichia coli* (ATCC 25922), analogously as in polypropylene nonwovens [65]. 

#### 5.2.9. Antifungal Activity

The antifungal activity of resulting samples was tested according to PN-EN 14119:2005 [95]. This standard indicates tests of antifungal activity on a *Chaetomium globosum* (ATCC 6205), analogously, as in PP nonwovens [65].

## Figures and Tables

**Table 1:** Quantitative results of EDS analysis of PLA, PLA–ALG–Na^+^, PLA–ALG–Cu^2+^-1 and PLA–ALG–Cu^2+^-2.

PLA							
Atom	C	O					
At. %	51.70	48.33
Std. dev.	0.11	0.11
**PLA** **–ALG** **–Na^+^**							
Atom	**C**	**O**	**Na**	**Ca**			
At. %	71.41	28.42	0.10	0.08			
Std. dev.	9.74	9.89	0.14	0.17			
**PLA** **–** **ALG** **–** **Cu^2+^-1**							
Atom	**C**	**O**	**Cu**	**Na**	**S**	**Cl**	**Ca**
At. %	55.26	40.95	0.92	0.78	0.02	1.93	0.15
Std. dev.	1.26	2.59	1.26	1.10	0.04	0.65	0.21
**PLA** **–ALG** **–Cu^2+^-2**							
Atom	**C**	**O**	**Cu**	**Na**	**S**	**Cl**	**Ca**
At. %	35.16	19.96	15.06	5.87	1.18	21.44	1.30
Std. dev.	8.66	2.67	1.53	0.89	0.59	5.23	0.75

**Table 2:** Copper concentration in polylactide/alginate/copper composite materials samples.

Sample Name	Cu Concentration (g/kg)
PLA	0.004
PLA–ALG–Na^+^	0.003
PLA–ALG–Cu^2+^-1	13.56
PLA–ALG–Cu^2+^-2	73.91

The results have been measured in triplicate and presented as a mean value.

**Table 3:** The Characteristic FTIR band frequencies determined for polylactide nonwoven, alginic acid sodium salt (ALG–Na^+^), ALG–Cu^2+^ complex, and PLA–ALG–Cu^2+^-2 composite [84,85,86,87,88,89,90,91,92].

PLA			ALG–Na^+^				ALG–Cu^2+^		PLA–ALG–Cu^2+^	
(ν/cm^−1^)	Int.^/a^	Assign. [84]	(ν/cm^−1^)	Int.^/a^	Lit. bands (ν/cm^−1^) [85,86,87,88,89,90,91]	Assign. /[Ref.][88]	(ν/cm^−1^)	Int.^/a^	(ν/cm^−1^)	Int.^/a^
					3350 ± 350	ν_s_ O–H	3750	0.013^/c^		
							3345	0.09^/c^		
			3200	0.14^/b^		ν_s_ O–H			3200	0.01
							3164	0.05		
2997	0.01	ν_as_ CH_3_								
2947	0.01	ν_s_ CH_3_								
					2926 ± 1	ν_s_ C–H			2890	0.01
			2897	0.01			2886	0.02	2886	0.01
							2389	0.02		
									2341	0.01
			2322	0.01			2300	0.02	2337	0.01
									2265	0.01
									2270	0.01
			2216	0.01					2205	0.005
			2134	0.01					2152	0.01
									2111	0.01
			2021	0.01					2025	0.01
			1957	0.01					1946	0.01
							1833	0.02		
1760	0.07	ν C=O							1755	0.01
					1650 [86]	ν_as_ COO^–^	1690	0.02		
					1614 [85,86]	ν_as_ COO^–^				
			1588	0.03			1581	0.09	1588	0.02
1452	0.02	δ_as_ CH_3_								
					1417 [85,86]	δ C–H; ν_s_ COO^–^			1411	0.02
			1400	0.02						
1388-1368	0.02	δ_s_CH_3_								
1368-1360	0.02	δ_1_ CH+δ_s_CH_3_								
1270	0.02	δCH+νCOC								
					1301 (ms)	δ C–H				
			1295	0.02						
1130	0.07	r_as_CH_3_			1124 (s) [86]	ν C–O, ν C–C, δ C–C–C, ν_as_ C–O–C^/d^			1178	0.02
1100-1090	0.11	ν_s_ COC			1096 (s) [86]	ν C–O, ν C–C, δ C–C–O;			1073	0.03
1045	0.08	ν C–CH_3_			1034 (vs) [86]	ν_as_ C–O–C^/d^, ν C–O^/d^ ν C–C				
			1017	0.04			1013	0.03	1020	0.03
875–860	0.03	νC–COO							866	0.02
					826 (ms) [86]	δ C–O–C^/d^,δ C–C–C, δ C–C–O^/d^ δ C–C–H, δω O–H	810	0.03	810	0.02
760–740	0.03	δC=O			776 (w)	rb, δ C–C–H, δ C–C–O				
715–695	0.03	γC=O			703(ms)	rb				
			555	0.04		δω O–H			555	0.03

^a^ Band assignment: ν, stretching vibration; δ, deformation; sh, shoulder; s, symmetric; as, asymmetric; r, rocking; γ, out-of-plane bending mode; ω, wagging; rb, ring breathing. ^b^ Band intensity: The bands with absorbance values ≥0.005 are listed, and subsequently approximated to a second decimal place. In the literature data: s, strong; vs, very strong; ms, medium strong; w, weak. ^c^ Derived from water hydrogen–bonded O–H stretching vibrations [92]. ^d^ Glycosidic linkage.

**Table 4:** Specific surface area, total pore volume, and average pore diameter of the examined samples.

Sample Name	Specific Surface Area S_BET_ (m^2^/g)	Total Pore Volume V (cm^3^/g)	Average Pore Diameter D (nm)
PLA	0.2405 ± 0.0220	9.084⋅10^−4^	13.85
PLA–ALG–Na^+^	0.5548 ± 0.0452	1.581⋅10^−3^	10.54
PLA–ALG–Cu^2+^-1	0.8429 ± 0.0109	3.450⋅10^−3^	16.59
PLA–ALG–Cu^2+^-2	1.4280 ± 0.0220	4.661⋅10^−3^	13.26

**Table 5:** UPF values of modified nonwoven fabric samples.

	PLA	PLA–ALG–Na^+^	PLA–LG–Cu^2+^-1	PLA–ALG–Cu^2+^-2
UPF	7.23	12.35	13.97	43.28
average %T, λ = 290–400 nm	16.46	10.63	9.03	4.43

The results have been measured in triplicate and presented as a mean value with ± deviation approximately 6%.

**Table 6:** Results of tests on the antibacterial activity of polylactide/alginate/copper (PLA–ALG–Cu^2+^) on the basis of standards EN–ISO 20645:2006 [94].

Sample Name	Bacterial Average Inhibition Zone (mm)	
	*Escherichia coli*	*Staphylococcus aureus*
PLA	0	0
PLA–ALG–Na^+^	0	0
PLA–ALG–Cu^2+^−1	3	2
PLA–ALG–Cu^2+^−2	3	4

Concentration of inoculum (bacterial suspension). Amount of live bacteria: *Escherichia coli*: CFU/mL = 1.8 × 10^8^ and *Staphylococcus aureus*: CFU/mL = 1.6 × 10^8^.

**Table 7:** The antifungal activities and growth inhibition effects of PLA–ALG–Cu^2+^-1/2 composites.

Sample Name	Fungal Average Inhibition Zone (mm)		
	*Aspergillus niger*	*Chaetomium globosum*	Visual Evaluation (Magnification 50×)
PLA	0	0	Visible growth on sample surface
PLA–ALG–Na^+^	0	0	
PLA–ALG–Cu^2+^−1	3	3	No visible growth on sample surface
PLA–ALG–Cu^2+^−2	3	3	

Concentration of inoculum, number of fungal spores in 1 mL: *Aspergillus niger*: CFU/mL = 2.9 × 10^6^, *Chaetomium globosum*: CFU/mL = 2.3 × 10^6^.

**Table 8:** Melt-blown process conditions applied for preparation of polylactide (PLA) nonwovens.

Parameter	
Polymer yields	5 g/min
Mass per unit area of nonwovens	160 g/m^2^
Air flow rate	7–8 m^3^/h
Temperature of the extruder: zone 1	195 °C
Temperature of the extruder: zone 2	245 °C
Temperature of the extruder: zone 3	260 °C
Head temperature	260 °C
Air heater temperature	260 °C

**Table 9:** Assignments of samples and composition of the coating components of poly(lactide) nonwoven samples surface modifier (%).

Assignments for Composites and Their Components	Coating Components (%)		
	Sodium Alginate Solution (ALG–Na^+^)	Copper(II) Chloride Solutions (CuCl_2_)	
	0, 5%	5%	10%
PLA	–	–	–
PLA–ALG–Na^+^	+	–	–
PLA–ALG–Cu^2+^-1	+	+	–
PLA–ALG–Cu^2+^-2	+	–	+

## References

[1] Garlotta D. (2001). A literature review of poly (lactic acid). J. Polym. Environ..

[2] Auras R., Lim L.T., Selke S.E.M., Tsuji H. (2010). Poly (Lactic Acid): Synthesis, Structures, Properties, Processing, and Applications.

[3] Bordes P., Pollet E., Avérous L. (2009). Nano–biocomposites: Biodegradable polyester/nanoclay systems. Prog. Polym. Sci..

[4] Park S.B., Lih E., Park K.S., Joung Y.K., Han D.K. (2017). Biopolymer–based functional composites for medical applications. Prog. Polym. Sci..

[5] Koh J.J., Zhang X., He C. (2018). Fully biodegradable Poly(lactic acid)/Starch blends: A review of toughening strategies. Int. J. Biol. Macromol..

[6] Granados–Hernández M.V., Serrano–Bello J., Montesinos J.J., Alvarez–Gayosso C., Medina–Velázquez L.A., Alvarez–Fregoso O., Alvarez–Perez M.A. (2018). In vitro and in vivo biological characterization of poly (lactic acid) fiber scaffolds synthesized by air jet spinning. J. Biomed. Mater. Res. B Appl. Biomater..

[7] Alex A., Ilango K.N., Ghosh P. (2018). Comparative role of chain scission and solvation in the biodegradation of polylactic acid (PLA). J. Phys. Chem. B.

[8] Nofar M., Sacligil D., Carreau P.J., Kamal M.R., Heuzey M.-C. (2019). Poly (lactic acid) blends: Processing, properties and applications. Int. J. Biol. Macromol..

[9] Rogovina S.Z., Aleksanyan K.V., Vladimirov L.V., Berlin A.A. (2019). Biodegradable polymer materials based on polylactide. Russ. J. Phys. Chem. B.

[10] Kliem S., Kreutzbruck M., Bonten C. (2020). Review on the biological degradation of polymers in various environments. Materials.

[11] Olewnik–Kruszkowska E., Burkowska–But A., Tarach I., Walczak M., Jakubowska E. (2020). Biodegradation of polylactide–based composites with an addition of a compatibilizing agent in different environments. Int. Biodeterior. Biodegrad..

[12] Lasprilla A.J.R., Martinez G.A.R., Lunelli B.H., Jardini A.L., Filho R.M. (2012). Poly–lactic acid synthesis for application in biomedical devices—A review. Biotechnol. Adv..

[13] Zhou J., Han S., Dou Y., Lu J., Wang C., He H., Li X., Zhang J. (2013). Nanostructured poly(l–lactide) matrix as novel platform for drug delivery. Int. J. Pharm..

[14] Pandey A.K., Dwivedi A.K. (2019). Recent advancement in wound healing dressing material. Int. J. Pharm. Sci. Res..

[15] Sharif A., Mondal S., Hoque M.E. (2019). Polylactic acid (PLA)–based nanocomposites: Processing and properties. Bio–based Polymers and Nanocomposites: Preparation, Processing, Properties & Performance.

[16] Alam F., Shukla V.R., Varadarajan K.M., Kumar S. (2020). Microarchitected 3D printed polylactic acid (PLA) nanocomposite scaffolds for biomedical applications. J. Mech. Behav. Biomed. Mater..

[17] Essa D., Kondiah P.P.D., Choonara Y.E., Pillay V. (2020). The Design of poly(lactide–co–glycolide) nanocarriers for medical applications. Front. Bioeng. Biotechnol..

[18] Wu D., Spanou A., Diez–Escudero A., Persson C. (2020). 3D–printed PLA/HA composite structures as synthetic trabecular bone: A feasibility study using fused deposition modeling. J. Mech. Behav. Biomed. Mater.

[19] Barceloux D.G., Barceloux D. (1999). Copper. J. Toxicol. Clin. Toxicol..

[20] Gaetke L.M., Chow–Johnson H.S., Chow C.K. (2014). Copper: Toxicological relevance and mechanisms. Arch. Toxicol..

[21] Scopus Base 5476 Documents Results on Antibacterial Copper. https://www-1scopus-1com-10000147v00b7.han.p.lodz.pl/results/results.uri?numberOfFields=0&src=s&clickedLink=&edit=&editSaveSearch=&origin=searchbasic&authorTab=&affiliationTab=&advancedTab=&scint=1&menu=search&tablin=&searchterm1=antibacterial+metallic+copper&field1=TITLE_ABS_KEY&dateType=Publication_Date_Type&yearFrom=Before+1960&yearTo=Present&loadDate=7&documenttype=All&accessTypes=All&resetFormLink=&st1=antibacterial+metallic+copper&st2=&sot=b&sdt=b&sl=44&s=TITLE–ABS–KEY%28antibacterial+metallic+copper%29&sid=f8209a25a17607eae8dff3520803ccdf&searchId=f8209a25a17607eae8dff3520803ccdf&txGid=19f07020ab9ba0c00ce0a023d21c5278&sort=plf–f&originationType=b&rr=.

[22] Saidin S., Jumat M.A., Mohd Amin N.A.A., Saleh Al–Hammadi A.S. (2021). Organic and inorganic antibacterial approaches in combating bacterial infection for biomedical application. Mater. Sci. Eng. C.

[23] Sthoer A., Hladılkova J., Lund M., Tyrode E. (2019). Molecular insight into carboxylic acid–alkali metal cations interactions: Reversed affinities and ion–pair formation revealed by non–linear optics and simulations. Phys. Chem. Chem. Phys..

[24] Draget K.L., Taylor C. (2011). Chemical, physical and biological properties of alginates and their biomedical implications. Food Hydrocoll..

[25] Polyzois G.L., Andreopoulos A.G. (1985). Stability of some soluble alginate solutions. Biomaterials.

[26] Achmon Y., Dowdy F.R., Simmons C.W., Zohar–Perez C., Rabinovitz Z., Nussinovitch A. (2019). Degradation and bioavailability of dried alginate hydrocolloid capsules in simulated soil system. J. Appl. Polym. Sci..

[27] Zhang C., Show P.L., Ho S.H. (2019). Progress and perspective on algal plastics—A critical review. Bioresour. Technol..

[28] Zhang W., Xia X., Zhang Z. (2019). Alginate lyase of a novel algae fermentation strain. Chem. Biochem. Eng. Q..

[29] Sun H., Gao L., Xue C., Mao X. (2020). Marine–polysaccharide degrading enzymes: Status and prospects. Compr. Rev. Food Sci. Food Saf..

[30] Lee K.Y., Mooney D.J. (2012). Alginate: Properties and biomedical applications. Prog. Polym. Sci..

[31] Pawar S.N., Edgar K.J. (2012). Alginate derivatization: A review of chemistry, properties and applications. Biomaterials.

[32] Venkatesan J., Bhatnagar I., Manivasagan P., Kang K.H., Kims K.A. (2015). Alginate composites for bone tissue engineering: A review. Int. J. Biol. Macromol..

[33] Fernando I.P.S., Kim D., Nah J.W., Jeon Y.J. (2019). Advances in functionalizing fucoidans and alginates (bio)polymers by structural modifications: A review. Chem. Eng. J..

[34] Kuznetsova T.A., Andryukov B.G., Besednova N.N., Zaporozhets T.S., Kalinin A.V. (2020). Marine algae polysaccharides as basis for wound dressings, drug delivery, and tissue engineering: A review. J. Mar. Sci. Eng..

[35] Yokoyama F., Achife C.E., Takahira K., Yamashita Y., Monobe K., Kusano F., Nishi K. (1992). Morphologies of oriented alginate gels crosslinked with various divalent metal ions. J. Macromol. Sci., Part B.

[36] García–Torres J., Gispert C., Gómez E., Vallés E. (2015). Alginate electrodeposition onto three–dimensional porous Co–Ni films as drug delivery platforms. Phys. Chem. Chem. Phys..

[37] Pistone S., Qoragllu D., Smistad G., Hiorth M. (2015). Formulation and preparation of stable cross–linked alginate–zinc nanoparticles in the presence of a monovalent salt. Soft Matter.

[38] Pérez–Madrigal M.M., Torras J., Casanovas J., Haring M., Alemán C., Díaz D.D. (2017). Paradigm shift for preparing versatile M^2+^–free gels from unmodified sodium alginate. Biomacromolecules.

[39] Frígols B., Martí M., Salesa B., Hernández–Oliver C., Aarstad O., Teialeret Ulset A.S., Sӕtrom G.I., Aachmann F.L., Serrano–Aroca A. (2019). Graphene oxide in zinc alginate films: Antibacterial activity, cytotoxicity, zinc release, water sorption/diffusion, wettability and opacity. PLoS ONE.

[40] Munhoz D.R., Bernardo M.P., Malafattia J.M., Moreira F.K.V., Mattoso L.H.C. (2019). Alginate films functionalized with silver sulfadiazine–loaded [Mg–Al] layered double hydroxide as antimicrobial wound dressing. Int. J. Biol. Macromol..

[41] Salesa B., Martí M., Frígols O.B., Serrano–Aroca A. (2019). Carbon nanofibers in pure form and in calcium alginate composites films: New cost–effective antibacterial biomaterials against the life–threatening multidrug–resistant Staphylococcus epidermidis. Polymers.

[42] Zahran M., Marei A.H. (2019). Innovative natural polymer metal nanocomposites and their antimicrobial activity. Int. J. Biol. Macromol..

[43] Susilowati E., Masykuri M., Hastuti B. (2020). Preparation of nanocomposite silver–chitosan–alginate film as antibacterial material. J. Phys. Conf. Ser..

[44] Porter G.C., Schwass D.R., Tompkins G.R., Bobbala S.K.R., Medlicott N.J., Meledandri C.J. (2021). AgNP/Alginate Nanocomposite hydrogel for antimicrobial and antibiofilm applications. Carbohyd. Polym..

[45] Cataldo S., Gianguzza A., Pettignano A., Villaescusa I. (2013). Mercury(II) removal from aqueous solution by sorption onto alginate, pectate and polygalacturonate calcium gel beads. A kinetic and speciation based equilibrium study. React. Funct. Polym..

[46] Shang Y., Yu X. (2015). Screening of algae material as a filter for heavy metals in drinking water. Algal Res..

[47] Fuks L., Herdzik–Koniecko I., Polkowska–Motrenko H., Oszczak A. (2018). Novel procedure for removal of the radioactive metals from aqueous wastes by the magnetic calcium alginate. Int. J. Environ. Sci. Technol..

[48] Ali A.M., Husson J., Monney S., Franchi M., Knorr M., Euvrard M. (2019). Biosorption of Pb(II) ions from aqueous solution using alginates extracted from Djiboutian seaweeds and deposited on silica particles. Pure Appl. Chem..

[49] Biswas S., Meikap B.C., Sen T.K. (2019). Adsorptive removal of aqueous phase copper (Cu^2+^) and nickel (Ni^2+^) metal ions by synthesized biochar–biopolymeric hybrid adsorbents and process optimization by Response Surface Methodology (RSM). Water Air Soil Pollut..

[50] Borgiallo A., Rojas R. (2019). Reactivity and heavy metal removal capacity of calcium alginate beads loaded with Ca–Al layered double hydroxides. Chem. Eng..

[51] Li J., He J., Huang Y. (2017). Role of alginate in antibacterial finishing of textiles. Int. J. Biol. Macromol..

[52] Grace M., Chand N., Bajpai S.K. (2009). Copper alginate–cotton cellulose (CACC) fibers with excellent antibacterial properties. J. Eng. Fiber. Fabr..

[53] Bajpai S.K., Bajpai M., Sharma L. (2012). Copper nanoparticles loaded alginate–impregnated cotton fabric with antibacterial properties. J. Appl. Polym. Sci..

[54] Heliopoulos N.S., Kouzilos G.N., Giarmenitis A.I., Papageorgiou S.K., Stamatakis K., Katsaros F.K. (2020). Viscose fabric functionalized with copper and copper alginate treatment toward antibacterial and UV blocking properties. Fibers Polym..

[55] Heliopoulos N.S., Papageorgiou S.K., Galeou A., Favvas E.P., Katsaros F.K., Stamatakis K. (2013). Effect of copper and copper alginate treatment on wool fabric. Study of textile and antibacterial properties. Surf. Coat. Technol..

[56] Scopus Base 12814 Documents Results on Alginates. https://www-1scopus-1com-10000145w03fc.han.p.lodz.pl/results/results.uri?numberOfFields=0&src=s&clickedLink=&edit=&editSaveSearch=&origin=searchbasic&authorTab=&affiliationTab=&advancedTab=&scint=1&menu=search&tablin=&searchterm1=alginates&field1=TITLE_ABS_KEY&dateType=Publication_Date_Type&yearFrom=Before+1960&yearTo=Present&loadDate=7&documenttype=All&accessTypes=All&resetFormLink=&st1=alginates&st2=&sot=b&sdt=b&sl=24&s=TITLE–ABS–KEY%28alginates%29&sid=31a31ef4754f2b4287c807b35585f4eb&searchId=31a31ef4754f2b4287c807b35585f4eb&txGid=db81be6eb51e1f2dee457d6275b329e1&sort=plf–f&originationType=b&rr=.

[57] Scopus Base 3534 Documents Results on Alginate Composites. https://www-1scopus-1com-10000145w03fd.han.p.lodz.pl/results/results.uri?numberOfFields=0&src=s&clickedLink=&edit=&editSaveSearch=&origin=searchbasic&authorTab=&affiliationTab=&advancedTab=&scint=1&menu=search&tablin=&searchterm1=alginate++composites+&field1=TITLE_ABS_KEY&dateType=Publication_Date_Type&yearFrom=Before+1960&yearTo=Present&loadDate=7&documenttype=All&accessTypes=All&resetFormLink=&st1=alginate++composites+&st2=&sot=b&sdt=b&sl=36&s=TITLE–ABS–KEY%28alginate++composites+%29&sid=49cbf4a20bebe6548c33dbebccd109e8&searchId=49cbf4a20bebe6548c33dbebccd109e8&txGid=b35ba7e690e08ac59a51dd1904037cab&sort=plf–f&originationType=b&rr==.

[58] Scopus Base 1282 Documents Results on Alginate Hybrids. https://www-1scopus-1com-10000145w03fd.han.p.lodz.pl/results/results.uri?numberOfFields=0&src=s&clickedLink=&edit=&editSaveSearch=&origin=searchbasic&authorTab=&affiliationTab=&advancedTab=&scint=1&menu=search&tablin=&searchterm1=alginate+hybrids++&field1=TITLE_ABS_KEY&dateType=Publication_Date_Type&yearFrom=Before+1960&yearTo=Present&loadDate=7&documenttype=All&accessTypes=All&resetFormLink=&st1=alginate+hybrids++&st2=&sot=b&sdt=b&sl=33&s=TITLE–ABS–KEY%28alginate+hybrids++%29&sid=49cbf4a20bebe6548c33dbebccd109e8&searchId=49cbf4a20bebe6548c33dbebccd109e8&txGid=b35ba7e690e08ac59a51dd1904037cab&sort=plf–f&originationType=b&rr==.

[59] Xua W., Shenc R., Yana Y., Gao J. (2017). Preparation and characterization of electrospun alginate/PLA nanofibers as tissue engineering material by emulsion eletrospinning. J. Mech. Behav. Biomed. Mater..

[60] Yang M., Yang T., Jia J., Lu T., Wang H., Yan X., Wang L., Yu L., Zhao Y. (2018). Fabrication and characterization of DDAB/PLA alginate composite microcapsules as single–shot vaccine. RSC Adv..

[61] Pandey G., Chaudhari R., Joshi B., Choudhary S., Kaur J., Joshi A. (2019). Fluorescent biocompatible platinum–porphyrin–doped polymeric hybrid particles for oxygen and glucose biosensing. Sci. Rep..

[62] Kudzin Z.H., Kudzin M.H., Drabowicz J., Stevens C.V. (2011). Aminophosphonic acids–phosphorus analogues of natural amino acids. Part 1: Syntheses of α–aminophosphonic acids. Curr. Org. Chem..

[63] Drabowicz J., Jordan F., Kudzin M.H., Kudzin Z.H., Stevens C.V., Urbaniak P. (2016). Reactivity of aminophosphonic acids. Oxidative dephosphonylation of 1–aminoalkylphosphonic acids by aqueous halogens. Dalton Trans..

[64] Kudzin Z.H., Depczyński R., Kudzin M.H., Łuczak J., Drabowicz J. (2007). 1–(N–Trifluoroacetylamino)alkylphosphonic acids: Synthesis and properties. Amino Acids.

[65] Kudzin M.H., Mrozińska Z., Walawska A., Sójka–Ledakowicz J. (2019). Biofunctionalization of textile materials. 1. Biofunctionalization of poly(propylene) (PP) nonwovens fabrics by Alafosfalin. Coatings.

[66] Kudzin M.H., Mrozińska Z. (2020). Biofunctionalization of textile materials. 2. Antimicrobial modification of poly(lactide) (PLA) nonwoven fabrics by fosfomycin. Polymers.

[67] Kudzin M.H., Mrozińska Z. (2020). Biofunctionalization of textile materials.3. Biofunctionalization of poly (lactide) (PLA) nonwovens fabrics by KI. Coatings.

[68] Sójka–Ledakowicz J., Kudzin M.H. (2014). Effect of plasma modification on the chemical structure of a polyethylene terephthalate fabrics surface. Fibres Text. East. Eur..

[69] Kudzin M.H., Mrozinska Z., Kaczmarek A., Lisiak–Kucinska A. (2020). Deposition of copper on poly (lactide) non–woven fabrics by magnetron sputtering–fabrication of new multi–functional, antimicrobial composite. Materials.

[70] Kudzin M.H., Kaczmarek A., Mrozińska Z., Olczyk J. (2020). Deposition of copper on polyester knitwear fibers by a magnetron sputtering system. Physical properties, and evaluation of antimicrobial response of new multi–functional composite materials. Appl. Sci..

[71] Guo X., Wang Y., Qin Y., Shen P., Peng Q. (2020). Structures, properties and application of alginic acid: A review. Int. J. Biol. Macromol..

[72] Lu L., Liu X., Qian L., Tong Z. (2003). Sol–gel transition in aqueous alginate solutions induced by cupric cations observed with viscoelasticity. Polym. J..

[73] Hernandez R., Sacristan J., Mijangos C. (2010). Sol/Gel Transition of aqueous alginate solutions induced by Fe^2+^ cations. Macromol. Chem. Phys..

[74] Brus B., Urbanova M., Czernek J., Pavelkova M., Kubova K., Vyslouzil J., Abbrent S., Konefal R., Horsky J., Vetchy D. (2017). Structure and dynamics of alginate gels cross–linked by polyvalent ions probed via solid state NMR spectroscopy. Biomacromolecules.

[75] Zhou Q., Kang H., Bielec M., Wua X., Cheng Q., Wei W., Dai H. (2018). Influence of different divalent ions cross–linking sodium alginate–polyacrylamide hydrogels on antibacterial properties and wound healing. Carbohyd. Polym..

[76] Yang N., Wang R., Rao P., Yan L., Zhang W., Wang J., Chai F. (2019). The fabrication of calcium alginate beads as a green sorbent for selective recovery of Cu(II) from metal mixtures. Crystals.

[77] Wang Z.Y., Zhang Q.Z., Konno M., Saito S. (1993). Sol–gel transition of alginate solution by the addition of various divalent cations: ^13^C–NMR spectroscopic study. Biopolymers.

[78] Tiwari A., Tiwari R., Bajpai A.K. (2009). Dynamic and equilibrium studies on adsorption of Cu(II) ions onto biopolymeric cross–linked pectin and alginate beads. J. Dispers. Sci. Technol..

[79] Brinza L., Geraki K., Cojocaru C., Holdt S.L., Neamtu M. (2019). Baltic Fucus vesiculosus as potential bio–sorbent for Zn removal: Mechanism insight. Chemosphere.

[80] Pan L., Wang Z., Zhao X., He H. (2019). Efficient removal of lead and copper ions from water by enhanced strength–toughness alginate composite fibers. Int. J. Biol. Macromol..

[81] Li S.S., Song Y.L., Yang H.R., An Q.D., Xiao Z.Y., Zhai S.R. (2020). Modifying alginate beads using polycarboxyl component for enhanced metal ions removal. Int. J. Biol. Macromol..

[82] Lucaci A.R., Bulgariu D., Popescu M.C., Bulgariu L. (2020). Adsorption of Cu(II) ions on adsorbent materials obtained from marine red algae *Callithamnion corymbosum* sp. oa. Water.

[83] The Perkin–Elmer Corporation (1996). Analytical Methods for Atomic Absorption Spectroscopy.

[84] Kister G., Cassanas G., Vert M. (1998). Effects of morphology, conformation and configuration on the IR and Raman spectra of various poly (lactic acid)s. Polymer.

[85] Lawrie G., Keen I., Drew B., Chandler–Temple A., Rintoul L., Fredericks P., Grøndahl L. (2007). Interactions between alginate and chitosan biopolymers characterized using FTIR and XPS. Biomacromolecules.

[86] Leal D., Matsuhiro B., Rossi M., Caruso F. (2008). FT–IR spectra of alginic acid block fractions in three species of brown seaweeds. Carbohyd. Res..

[87] Lim S.F., Zheng Y.M., Zou S.W., Chen J.P. (2008). Characterization of copper adsorption onto an alginate encapsulated magnetic sorbent by a combined FT–IR, XPS, and mathematical modeling study. Environ. Sci. Technol..

[88] Cardenas–Jiron G., Leal D., Matsuhiro B., Osorio–Roman I.O. (2011). Vibrational spectroscopy and density functional theory calculations of poly– D–mannuronate and heteropolymeric fractions from sodium alginate. J. Raman Spectrosc..

[89] Díaz–Visurraga J., Daza C., Pozo C., Becerra A., von Plessing C., García A. (2012). Study on antibacterial alginate–stabilized copper nanoparticles by FT–IR and 2D–IR correlation spectroscopy. Int. J. Nanomed..

[90] Nastaj J., Przewłocka A., Rajkowska–Myśliwiec M. (2016). Biosorption of Ni(II), Pb(II) and Zn(II) on calcium alginate beads: Equilibrium, kinetic and mechanism studies. Pol. J. Chem. Technol..

[91] Fertah M., Belfkira A., Dahmane E.M., Taourirte M., Brouillette F. (2017). Extraction and characterization of sodium alginate from Moroccan Laminaria digitata brown seaweed. Arab. J. Chem..

[92] Falk M., Ford T.A. (1966). Infrared spectrum and structure of liquid water. Can. J. Chem..

[93] International Organization for Standardization (2002). Textiles. Solar UV Protective Properties. Method of Test for Apparel Fabrics.

[94] International Organization for Standardization (2006). Determination of Antibacterial Activity—Agar Diffusion Plate Test.

[95] International Organization for Standardization (2005). Testing of Textiles. Evaluation of the Action of Microfungi. Visual Method.

